# Human‐induced evolution: Signatures, processes and mechanisms underneath anthropogenic footprints on natural systems

**DOI:** 10.1111/eva.13305

**Published:** 2021-10-27

**Authors:** Miguel Baltazar‐Soares, Kristien I. Brans, Christophe Eizaguirre

**Affiliations:** ^1^ Department of Biology University of Turku Turku Finland; ^2^ Department of Biology Laboratory of Aquatic Ecology, Evolution and Conservation KU Leuven Leuven Belgium; ^3^ School of Biological and Chemical Sciences Queen Mary University of London London UK

**Keywords:** behaviour, genetics and epigenetics, human‐induced evolution, life history traits

## Abstract

The impact of human activities on the global environment has increased to such an extent that the current geological era has been coined the Anthropocene. Studies dedicated to understanding the evolutionary consequences of human‐induced selection on all levels of diversity (species, populations, traits, genes) provide direct knowledge about the mechanisms underlying species' responses and their evolutionary potential. A better understanding of the effects of human‐induced selection is needed to leverage evolved mechanisms to develop appropriate conservation programmes to guarantee the maintenance of healthy systems. In this special issue, we focus on different types of human‐mediated selection pressures, from the direct harvesting of individuals (e.g. hunting, fishing), to the more pervasive effects of climate change. Contributions highlight the diversity of human‐induced selection pressures ranging from fisheries, trophy‐hunting, poaching and domestication to climate change, and pollution. With those, we question whether there are parallel evolutionary solutions across fisheries systems, whether hunting pressures alter population dynamics and population structure, and whether climate change is an evolutionary dead‐end. The contributions reflect the direction of travel of the field and the solutions to mitigate the impact of human activities.

## 1 INTRODUCTION

Earth's geological timescale has been historically defined by natural events that substantially transformed the global environment (Lewis & Maslin, 2015). Transitions, such as those marking the Permian–Triassic boundary or the Cretaceous–Paleogene boundary, are geologically established events of our planet's history characterized by extensive change in the Earth's biota (Erwin, 1998; Jablonski, 1986). Nowadays, it is widely acknowledged that the exponential increase of human activities has had a comparable impact, and as such, the current times have been coined the Anthropocene (Ceballos et al., 2015; Crutzen & Stoermer, 2000; Lewis & Maslin, 2015). Anthropogenically driven environmental impacts encompass, for instance, pronounced changes in biogeochemistry such as the alteration of the carbon cycle because of excessive burning of fossil fuel into the atmosphere, enhancing greenhouse effects (IPCC, 2021; Raupach & Canadell, 2010; Summerhayes & Zalasiewicz, 2018; Vitousek et al., 1997). Specifically to biological systems, human activities have caused a series of local extinctions and the selective extirpation of large‐bodied animals from terrestrial and marine environments (Barnosky et al., 2011; Turvey & Crees, 2019). Arguably, human‐mediated selection constitutes one of the greatest and most pervasive selective forces on Earth (Hendry et al., 2017; Palumbi, 2001) changing at a pace that requires rapid evolution of adaptive responses.

This Special Issue stems from a symposium we organized at the 2019 European Society of Evolutionary Biology (ESEB) meeting in Turku, Finland, that focused on detailing the emergence of evolutionary responses to a broad range of human activities. Here, we expanded the scope to include a set of timely reviews and perspectives. The overarching goal of this Special Issue is to beacon discussions and raise awareness on scenarios of human‐induced evolution, state‐of‐the‐art methods and overall strategies to understand and characterize signatures of human‐induced evolution. Here, we define human‐induced evolution as emergent evolutionary changes either on heritable traits or on their genetic and epigenetic basis as responses to direct or indirect pressures imposed by human activities. Among the earlier and better‐established examples of human‐induced selection for desirable traits is animal and plant domestication, which has enabled the transition from nomadic hunter‐gathering societies to a sedentary, agriculture‐based, livelihood (Goudie, 2018). Overall, our socio‐economic development is intertwined with increasing pressures on the environment, ranging from local or regional effects, such as shaping river courses during the Bronze Age, to more global impacts, such as fossil fuel combustion that prompted the Industrial Revolution (Leigh et al., 2019; Zalasiewicz et al., 2011). Because contemporary anthropogenic activities impose strong ecological selective pressures, there are arguably more suitable scenarios to observe real‐time evolution in natural settings than environments disturbed by human activities. Identifying evolutionary responses fuelled by our actions, including the quantification of their magnitude across space and time, is critical to estimate long‐term consequences of human‐induced evolution on biodiversity and ecosystem functioning (Lambert & Donihue, 2020). Understanding the processes underlying evolutionary responses will also facilitate the development of appropriate mitigation strategies to reduce detrimental impacts of human activities on natural systems (Ottenburghs, 2021).

### 1.1 Human‐induced evolution as natural experiments

Translocations, defined as the active transport of specimens across geographically unconnected areas, is representative of the human ability to manipulate migration and gene flow (Heckwolf et al., 2021). In the past, translocations were necessary operations to maintain the comfort necessary to flourishing societies, such as the transport of domesticated dogs from the Old to the New World (Hofman & Rick, 2018). Currently, those activities are performed with different objectives that may range from restoring biodiversity of depleted habitats, as pest controls, or simply to enhance the aesthetics of urbanized areas. In the context of a conservation effort of whitefish species (*Coregonus* sp.) in Scottish lakes, Crotti et al. (2021) investigated six recently man‐made whitefish populations that originated from a translocation event to boost these fish's abundances. Results point out an overall lower genomic diversity and higher inbreeding among translocated populations were also associated with a high degree of differentiation among original and translocated populations. Differentiation was observed for (a) eco‐morphological traits associated with body shape, (b) DNA methylation patterns and (c) SNPs that revealed genomic selection in response to translocations. Overall, the species' evolutionary potential is apparently sufficient to overcome genetic bottlenecks (Crotti et al., 2021). The holistic approach undertook by Crotti et al. is the one needed to quantify the evolutionary potential of such species (Eizaguirre & Baltazar‐Soares, 2014). Another sort of human‐mediated species' movement relates to biological organisms that hold a symbiotic relationship with humans via domestication. The establishment and expansion of human settlements provide the opportunity for secondary contact between domesticated species and their wild counterparts (Ottenburghs, 2021). If reproductive isolation is incomplete, then hybridization between domesticated and wild species can occur (Grabenstein & Taylor, 2018; Hamilton & Miller, 2016). Capturing signatures of hybridization events is critical to maintain the integrity of wild specimens in the ever‐expanding era of the Anthropocene (Ottenburghs, 2021). In this context, genome scans are useful to reveal signatures of human‐driven hybridization as those can identify introgression patterns among admixed populations of domesticated and wild species (Pilot et al., 2021). Introgression from free‐ranging domestic dogs into populations of Eurasian wolves for example has shown that the transfer of genetic material might reduce Eurasian's wolf viability by genetic swamping. This effect further impact genetic diversity that is already negatively affected by genetic drift associated with small population sizes resulting from hunting and habitat loss (Pilot et al., 2021). The study provides new perspective to address conservation efforts of wolf's genetic integrity; namely, it advocates the maintenance of large population sizes as an important measure of management strategies in order to avoid swamping by dog‐derived variants in wolf's genome (Pilot et al., 2021). Because of the longstanding contact and dependence on human activities, farmed animals might pose a similar risk to natural populations upon reproduction with wild animals (Le Luyer et al., 2017; Leitwein et al., 2021). Hence, it is critical to identify and characterize hypothetical marks of human‐induced selection and whenever possible the effects on fitness‐related traits. Utilizing a whole genome bisulphite sequencing approach, Leitwein et al. (2021) showed that hatchery‐reared Coho salmon (*Oncorhynchus kisutch*) exhibit a distinct epigenetic make‐up, both at a chromosomal and at a localized level, from those born in the wild despite being genetically indistinguishable. The fact that those epigenetic marks persist through adulthood in germline cells opens room for transgenerational inheritance and the potential spread of human‐induced signatures among wild populations.

Overall, the prevalence of anthropogenic hybridization that threatens the viability of wild populations calls for more efforts in exploring and predicting the evolutionary trajectories and outcome of hybridization (McFarlane et al., 2020). Ottenburghs (2021) summarizes the state of the art in this field and calls for speciation genomics specialists to focus on past hybridization events to predict introgression patterns among recently interbred species. Genome‐wide scans associated with mapping into annotated reference genomes can enable identifying the genomic regions primarily introgressed, revolutionizing the development of diagnostic genetic markers underlying functional traits (Ottenburghs, 2021).

### 1.2 Antagonistic effects of human‐induced selection on behaviour and life history and impacts to population dynamics

In contrast to translocations or anthropogenic hybridization, harvesting activities such as hunting, or fishing exert a predictable selection because its strong intensity and direction are largely dictated by specific harvesting strategies (Allendorf & Hard, 2009; Hočevar & Kuparinen, 2021; Van de Walle et al., 2021). Consequently, harvesting re‐shapes selection regimes that populations have adapted to and can result in antagonistic phenotypic responses of fitness‐related life history traits (Heino et al., 2015; LaSharr et al., 2019). For instance, the use of nets with specific mesh size and standardized hook size renders fishing a size‐selection experiment that promotes faster development rates, thus selecting for early age at maturation (Hutchings & Kuparinen, 2020). Similarly, hunting of bighorn sheep (*Ovis canadensis*) with large horn size imposes a gradual reduction of male horn length, a secondary sexual trait (Pigeon et al., 2016). While our knowledge on possible antagonistic evolutionary responses to harvesting increases, the role of demographic factors and impacts of adaptive evolution on the population dynamics remain poorly understood. In this special issue, Crespel et al. (2021) undertook to experimentally simulate harvesting in groups of fish kept at different standardized densities to better understand the impact of fishing pressure. They particularly focused on social behaviours and their density‐dependence. Results suggest that while behavioural traits such as high aggressiveness and less sociability are selected for by trawling independently of population density, the heritability of behaviours associated with fish activity and exploration change according to population density. These evolutionary responses can negatively impact social cohesion thus acting against evolved antipredator behaviours of grouping while maximizing the chances of more solitary fish to encounter predators (Crespel et al., 2021). Overall, Crespel et al. showed that trawling adds other selective pressures to those exerted by mesh size. As such, the legal fishing frameworks should be expanded beyond the simplistic scope of size‐based regulations to enable fish to cope with human‐induced antagonistic selection pressures (Crespel et al., 2021).

Trophy hunting activities exert a similar pressure to that of fishing since it removes individuals with specific traits. This effect is further amplified if those traits are involved in sexual selection. Modelling the population dynamics of Brown bear (*Ursus arctos*) in Sweden exposed to four different hunting regimes, that is, no protection, only mothers are protected, mothers and dependent offspring are protected and entire family groups are protected, showed that reproductive traits such as litter size and annual probability for females to reproduce have a large impact on population growth (Van de Walle et al., 2021). This result is particularly evident when specific hunting regulations are designed to protect only mothers (Van de Walle et al., 2021). Furthermore, expected hunting‐induced selection on female reproductive traits increases selectivity to produce larger litters at younger ages with increasing hunting quotas. With this work, Van de Walle et al. opens research lines to investigate possible feedback of evolutionary changes in life history traits on population processes, perhaps similar to how fisheries‐induced evolution selects for early age at maturation strategies. Impact of intense harvesting is expected to propagate not only within populations of exploited species but also to the ecosystem. Indeed, evolved responses to human‐induced selection affects ecosystem functionality, particularly if keystone species are those targeted by selection (Hočevar & Kuparinen, 2021). Fisheries‐induced evolution provide scenarios where the propagation of evolutionary responses can be traced across marine systems and Hočevar and Kuparinen (2021) offered us a perspective of such by exploring potential pathways in which size selection in fisheries might have eco‐evolutionary repercussions at ecosystem level. By screening our current knowledge on the impact of fisheries‐induced evolution in marine food webs, Hočevar and Kuparinen (2021) explored how size truncation may induce shifts in ecological niches of harvested species, how a changed maturation schedule might affect the spawning potential and biomass flow, how changes in life histories can initiate trophic cascades, how the role of apex predators may be shifting, and whether fisheries‐induced evolution could codrive species to depletion and biodiversity loss.

Human‐induced responses that contrast those that emerged through natural selection are not exclusive to harvesting regimes. By investigating the effect of increased algal turbidity, Candolin et al. (2021) found that courtship behaviour of male sticklebacks (*Gasterosteus aculeatus*) is highly plastic and exposed to differential selection under eutrophic conditions. However, most of the plasticity appears to be maladaptive: turbid conditions lead males to spend more time by the nest, contrary to female preferred behaviour such as increased search activity and time spent by the female. Comparable changes in behaviours during the reproductive period were also observed in sea turtles (*Caretta caretta*) nesting in Zakynthos (Greece), whereby the presence of tourists forces the turtles to use suboptimal, slightly cooler waters, that slow‐down egg development (Schofield et al., 2021). The impact is a likely longer nesting period, which may result in lower hatching success, ultimately selecting for faster development with unknown consequences. The forced displacement of turtles was only visible because of the ban on travel related to the SARS‐CoV2 pandemic, which reduced the effects of tourist presence on turtle behaviours (Schofield et al., 2021).

Observations that anthropogenic actions exert a selective pressure in the opposite direction of natural selection implies that management strategies undertaken to mitigate human‐induced evolution should consider possible trade‐offs in populations already adapted to human pressure (Hočevar & Kuparinen, 2021; Van de Walle et al., 2021). Given that the reversibility of evolutionary changes remains largely unknown, it is perhaps advisable to acknowledge factors such as the heterogeneity of selective pressures across spatial and temporal scales prior to the implementation of uniform measures.

### 1.3 Pollution and multistressor environments of urbanized areas

Because of the immediate impact on the fitness and health of organisms, pollution is considered one of the major contributors of human‐induced evolution (Brans et al., 2021). Mining, fossil fuel usage and large‐scale application of pesticides release excessive amounts of nondegradable pollutants into the environment, both terrestrial and aquatic. High concentrations of chemicals or heavy metals disrupt metabolic pathways and biological functions, and as such, the pressure imposed is direct and directional for increasing tolerance. With that in mind, Calboli et al. (2021) performed a genome‐wide association study to investigate the genetic basis of mercury tolerance in sticklebacks (*Gasterosteus aculeatus*) collected along a gradient of polluted environments. Exploring an association between mercury in muscle tissue and genome‐wide genomic variants, Calboli et al. found localized genomic regions putatively involved in adaptation to mercury‐polluted environments. The study further identified outlier loci in regions known to be involved in spacing of pharyngeal teeth, probably suggesting a functional adaptive link, whether direct or indirect, between diet and pollution. While a strong selective pressure, pollution is seldom the unique stressor to which organismal populations of urbanized areas are subjected to (Brans et al., 2021). Through the exposure of clonal lineages of urban and rural *Daphnia magna* populations to acute toxicity (organophosphate pesticide chlorpyrifos) at two temperatures, Brans et al. (2021) showed that urban water flea populations have higher survival probabilities, independent of temperature regimes compared to those from rural origin. Resistance against toxic agents suggests the evolution of an adaptive response that could ultimately hinder pest‐control strategies and facilitate persistence of these organisms in urbanized environments. Because human impacts are relatively recent, reviving dormant stages of organisms collected from pristine environments prior the exposure to intense selection offer an interesting perspective to characterize evolutionary trajectories of those species (Weider et al., 2018). It can inform on timing of the response as well as the tolerance to variable selection pressure. By performing experiments on resurrected *Daphnia* populations that were either naïve or exposed to human impacts over time, Cuenca‐Cambronero et al. (2021) explored whether and how chemical pollution and eutrophication triggered adaptive responses. Results showed that the emergence of adaptive responses to multiple human‐induced stressors comes with added selection in the form of increased mortality and reduced fertility (Cuenca‐Cambronero et al., 2021).

In general, pollution is an added selection pressure to those already experienced by natural populations. Like many others, the intensification of human activities creates a directional selection pressure that species need to cope with. Whether the pace of the response is sufficient to enable their persistence is still unknown but hints that with a sufficiently large population size, gene flow and well‐managed policy programme, positive outcome are possible.

### 1.4 The impact of anthropogenic climate change—shifts on species distributions and connectivity

Temperature has been one of the most significant mediators of species' diversity and distribution on Earth over geological times. We now know that shifts either in temperature or in temperature‐dependent environmental variables have been involved in large‐scale extinction events, or in changes in distribution via retractions/expansions during the Quaternary's glacial cycles (Hewitt, 2000; Penn et al., 2018). With temperatures rising at unprecedented pace, anthropogenic climate change is bound to dictate large shifts in the distribution of contemporary organisms (Rosenzweig et al., 2008). Modelling distribution shifts is thus an active area of research that represents one possibility to predict responses of certain species. Among others, implications of a climate change scenario extend to intrinsic biological processes, such as phenology‐related aspects of flowering plant's life cycles, insects life history, or bird massive migration mediated by seasonality. Here, Lockley and Eizaguirre (2021) reflects over the impacts of anthropogenic climate change on species whose sex is determined by temperature. Temperature‐dependent sex determination (TSD) exists in over 400 species, where the incubation temperature mediates gonad development. Lockley and Eizaguirre (2021) reviewed the specific literature to explore predictions that under fast‐paced changing climate scenarios, TSD species might be confronted with skewed sex ratios and risk extinction if evolutionary responses do not emerge as quickly as temperature shifts (Lockley & Eizaguirre, 2021). Yet, they also uphold the suggestion that since species with TSD have experienced large‐scale climatic events across their evolutionary history, the species must have evolved mechanisms that buffer sex ratio bias, further, suggesting that maternal hormone transfer enables to resist the developmental transition from male to female in sea turtles as temperature increases. Jointly with evolutionary responses, the ability for depauperated populations to recover from environmental disturbances depends on the availability of migrants from unaffected locations. Reef systems are particularly vulnerable to climate change, with thermal stress causing bleaching events that might occur at very localized scales (Afiq‐Rosli et al., 2021). Hence, characterizing the connectivity across large geographic scales is critical to identify potential source populations and eventually perform restoration activities. Afiq‐Rosli et al. (2021) exemplified just that in a study that comprised the screen of over 30k SNPs obtained with NextRAD from nine populations of reef‐building corals (*Porites* sp. and *Pocillopora acuta*). The identification of fine‐scale population structure might provide solutions for management strategies that aim to repopulate bleached systems.

Overall, studies investigating the effects of climate change need to consider many elements. Firstly, species have survived past climatic events and therefore may have evolved still unknown mechanisms, which contribute to a species' evolutionary potential. This calls for mechanistic studies to contribute to studying the effect of climate change. Secondly, species may evolve partial tolerance to changes and species distribution shift need to consider that species may look for suboptimal conditions. Lastly, there is a clear role for evolutionary biologists to play to guide conservation efforts to leverage natural mechanisms to manage populations.

### 1.5 Evolutionary applications in the Anthropocene

The global impact of anthropogenic activities is likely to influence the evolutionary trajectories of all species on the planet. Strong and directional selective pressures imposed by human activities are bound to leave stark footprints on genomes and epigenomes, directing phenotypic responses/phenotypic space to edges that often mismatch those that have been shaped by natural selection over long periods. The breaking point is real and exists when the fitness advantage of naturally evolved responses clash with those of anthropogenically evolved responses (Hočevar & Kuparinen, 2021). Still, one may argue that the perception that humans have the capacity to significantly alter ecosystems is relatively recent. With the increasing empirical evidence on single species evolutionary responses to human activities, theoretical and experimental set‐ups have started to focus on potential long‐term effects on evolutionary trajectories and dynamics of impacted populations. Indeed, approaches that investigate how species' evolutionary responses of disturbed species can impact ecosystem services are mandatory to predict and mitigate effects of human‐induced evolution on population dynamics (Des Roches et al., 2021). Thus, perhaps the greatest challenge of nowadays applied evolutionary biology is whether we can predict evolutionary trajectories over long periods of anthropogenic‐driven environmental disturbance. To shed light over that topic, Coulson et al. (2021) brought forth a perspective to explore how environmental effects over multiple generations could be added to models that commonly rely only on additive genetic variation to predict evolutionary trajectories over a single generation. Because Coulson's et al. (2021) evolutionary explicit Integral Projection Models are built to decompose the dynamics of the covariance between phenotypic traits and absolute fitness from mean fitness as a function of environmental variation, those might be suitable to explore the fast paces of human‐induced environmental disturbance. In conclusion, human‐induced evolution manifest across several scenarios and signatures of evolutionary responses are becoming as ubiquitous as our presence or Earth. Considering that the signatures of evolutionary responses might propagate across generations, measures to tackle detrimental effects of human‐induced evolution must necessarily have in mind reversibility and thus impacts to fitness of disturbed populations.

## CONFLICT OF INTERESTS

The authors declare no conflict of interest.

## Data Availability

Does not apply to this manuscript.
